# Longitudinal cerebrospinal fluid biomarker measurements in preclinical sporadic Alzheimer's disease: A prospective 9-year study

**DOI:** 10.1016/j.dadm.2015.09.002

**Published:** 2015-10-09

**Authors:** Erik Stomrud, Lennart Minthon, Henrik Zetterberg, Kaj Blennow, Oskar Hansson

**Affiliations:** a Clinical Memory Research Unit, Department of Clinical Sciences, Malmö, Lund University, Skåne University Hospital, Malmö, Sweden; b Memory Clinic, Skåne University Hospital, Malmö, Sweden; c Clinical Neurochemistry Laboratory, Department of Psychiatry and Neurochemistry, Institute of Neuroscience and Physiology, Sahlgrenska Academy, University of Gothenburg, Mölndal, Sweden; d UCL Institute of Neurology, London, UK

**Keywords:** Dementia, Alzheimer's disease, Cognitive aging, Cerebrospinal fluid, Cohort studies, β-Amyloid_1–42_, Tau protein

## Abstract

**Introduction:**

Ascertainment of the pattern and temporal change of biomarkers in preclinical (asymptomatic) sporadic Alzheimer's disease (AD) will increase knowledge about early pathogenesis and facilitate interventional therapeutic trials.

**Methods:**

In this prospective longitudinal study, repeated cerebrospinal fluid (CSF) collections and cognitive evaluations were performed in cognitively healthy elderly individuals during a 9-year period.

**Results:**

Low CSF β-amyloid (Aβ)_42_ levels predicted subsequent development of clinical AD 9 years later. Noteworthy, one-third of individuals with pathologically low baseline Aβ_42_ levels remained cognitively intact during follow-up. No further decrease in Aβ_42_ was seen in those with low levels already at baseline.

**Discussion:**

CSF Aβ_42_ predicts sporadic AD at least 9 years before dementia onset and has plateaued already at this time. However, many individuals can harbor brain amyloid accumulation over a decade without signs of cognitive deterioration, which could implicate how CSF biomarkers are used to identify preclinical AD in future interventional therapeutic trials.

## 1 Introduction

The slowly progressive nature of Alzheimer's disease (AD) implies a long preclinical phase before onset of cognitive symptoms. Increasing evidence suggests that cerebral accumulation of β-amyloid (Aβ) can be detected 5–20 years before dementia onset in AD, when using cerebrospinal fluid (CSF) Aβ_42_ or amyloid positron emission tomography (PET) imaging [1], [2], [3], [4]. Important evidence comes from studies evaluating asymptomatic individuals with autosomal dominant forms of AD [1], [5]. To determine the temporal evolution of AD biomarkers during the early phases of sporadic AD, we need longitudinal studies with repeated biomarker assessments over 5–15 years covering the preclinical phases of AD. A few studies with repeated longitudinal biomarker assessments in cognitively healthy individuals have been published [6], [7], [8], but studies over extended periods, of >4 years, are still lacking.

Several studies imply that CSF can identify cognitively healthy elderly individuals that are at increased risk of subsequent development of cognitive decline [9], [10], [11], [12], [13], [14]. However, the frequency of false positive cases is still unclear. To address this, we need long-term follow-up cognitively healthy individuals with deviant CSF biomarkers.

In this prospective and longitudinal study, we investigated CSF biomarkers repeatedly over 9–10 years in individuals, who all were cognitively healthy at baseline. The cognitive performance and development of dementia were determined during the clinical follow-up. Great care was taken to minimize drop-out during the study.

## 2 Methods

### 2.1 Study design

The objective of this study was to model within-person neurodegenerative biomarker trajectories in preclinical AD using repeated assessments of CSF biomarkers and cognitive performance as well as to investigate the predictive ability of CSF biomarkers to identify future development of clinical dementia. It is a prospective, longitudinal, observational study on initially cognitively healthy elderly volunteers recruited through advertisement in year 2002 in the city of Malmö, Sweden [13], for the purpose to constitute a healthy control group in dementia studies. Individuals who responded were included in the study unless they fulfilled any of the prospectively set exclusion criteria. Baseline exclusion criteria were (1) subjective cognitive decline, (2) presence of mild cognitive impairment (MCI) or dementia, (3) mini-mental state examination (MMSE) [15] score of <27, and (4) presence of other morbidities possibly affecting cognitive status such as major depressive episode, ongoing alcohol abuse, and severs disorders of the central nervous system. Treatable and reversible diseases that could affect cognition were treated and did not lead to exclusion. Included participants were then followed longitudinally in approximately every third year with focus on cognitive performance and CSF measurements.

### 2.2 Subjects

**Fig. 1:**
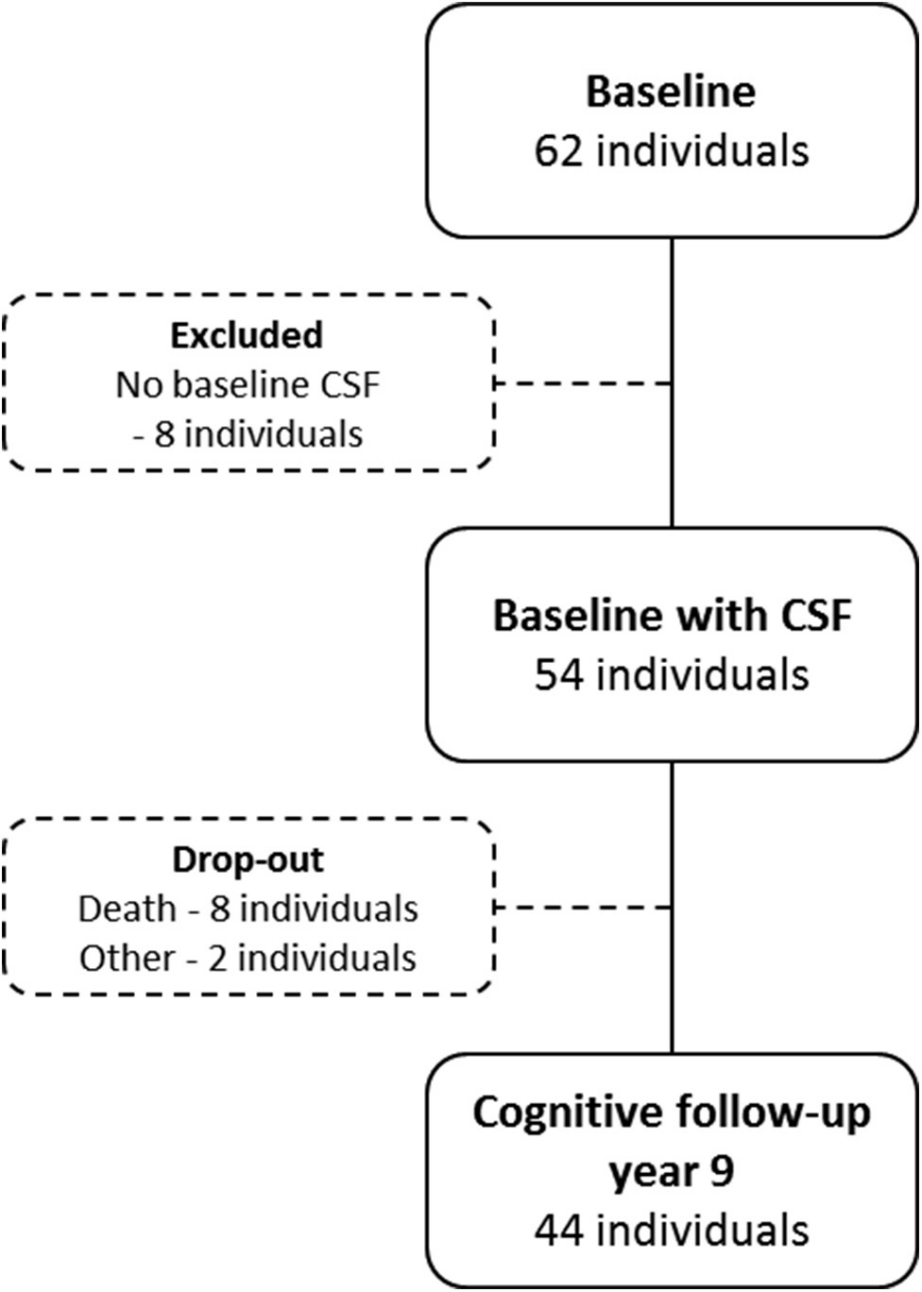
Flowchart of inclusion and drop-out in the study sample. Abbreviation: CSF, cerebrospinal fluid.

In total, 62 individuals could be recruited of which 54 performed baseline lumbar puncture and CSF collection. All participants also underwent comprehensive examination including physical, neurologic, and psychiatric evaluation, computed tomography (CT) of the brain, and cognitive testing at baseline. Cognitive follow-up was offered after 3, 5, and 9–10 years with renewed lumbar puncture after 5 and 9–10 years. Individuals with baseline CSF values and clinical cognitive follow-up after 9 years (n = 44) were included in the main analyses of the present study (Fig. 1). Only a handful of participants were evaluated after closer to 10 years at the last follow-up, whereas the overwhelming majority was evaluated after 9 years.

In the subgroup of participants who were not available for the 9-year follow-up visit (n = 10, Fig. 1), medical record was collected and antemortem cognitive follow-up performances in the study were evaluated in nine cases. In this subgroup, we found one individual who had developed MCI and the rest were cognitively normal at the last observation.

### 2.3 Cognitive evaluation

Cognitive testing included MMSE (all visits) [15], clock drawing test (all visits) [16], cube drawing (all visits) [17], delayed memory in Alzheimer's disease assessment scale cognitive subscale (ADAS-cog; baseline, follow-up years 5 and 9) [17], and a quick test (follow-up years 3, 5, and 9) [18]. At follow-up after 9 years, Stroop test [19], trail making test A and B [20], symbol digit modalities test [21], letter S fluency test (phonemic fluency) [22], animal fluency test (semantic fluency) [22], and month naming test (the task of naming the months backward as fast as possible starting with December) were also added. Delayed memory was scored as number of correctly recalled words, which gives higher scores when better delayed memory function.

Cognitive diagnosis was based on clinical evaluation by a physician experienced in dementia disorders and was later confirmed by a consensus group of experienced physicians. The consensus group was blinded to biomarker values and had only access to medical history, cognitive test results, and CT scan results. Diagnosis criteria used in regular clinical settings were applied, i.e. MCI [23], Alzheimer's dementia [24], vascular dementia [25], dementia with Lewy body (DLB) [26], and other dementia (OD) [27]. DLB and AD participants are studied together in this study because DLB patients often also have amyloid pathology next to their synucleinopathy [28].

### 2.4 Lumbar puncture and CSF analyses

Lumbar puncture was performed in a sitting position with CSF obtained from the L3/L4 or L4/L5 interspaces. All CSF were collected in plastic (polypropylene) tubes, gently mixed to avoid gradient effect. Samples were then centrifuged at 2000 × *g* at 4°C for 10 minutes to eliminate cells and other insoluble material. Pending biochemical analyses samples were immediately frozen and stored at −80°C without being thawed or refrozen.

Analysis of Aβ_42_, total tau (T-tau), and phosphorylated tau (P-tau) using xMAP technology (INNO-BIA AlzBio3 kit; Innogenetics, Ghent, Belgium) was performed using the same batch of reagents for each CSF acquisition. A large random sample of baseline and 5-year follow-up CSF was analyzed together with 9-year follow-up CSF to assure concordant assay values between all three analysis occasions. CSF values are given in nanograms per liter.

### 2.5 Ethics

The study was approved by the regional ethics committee at Lund University. All participants gave their written informed consent at baseline and at each follow-up.

### 2.6 Statistical analysis

IBM SPSS statistics version 22 was used for statistical analysis. Nonparametric tests were used because of the low number of cases and the nonnormal (Gaussian) distribution of CSF biomarker levels. Differences between diagnosis groups were calculated with Kruskal-Wallis test followed by Mann-Whitney U test when applicable. Mann-Whitney U test was used for dichotomized variables. Paired samples were analyzed using Wilcoxon signed-rank test. Cox regression models were set for prediction of AD and DLB as well as cognitive impairment. Baseline CSF levels and baseline delayed word recall score were added separately to the model with the following covariates: age, gender, and presence of apolipoprotein E (*APOE*) ε4 allele. Finally, a model including CSF levels, delayed word recall, and all covariates were created. Results are presented as hazard ratio (HR) with the 95% confidence interval (CI). For baseline CSF levels, standardized z-scores were used so that HR reflects change per one standard deviation (SD). Cox regression models were performed on all participants with baseline CSF measurements and at least one cognitive follow-up (n = 53), thus including the individuals that died during the follow-up period.

**Supplementary Fig. 1:**
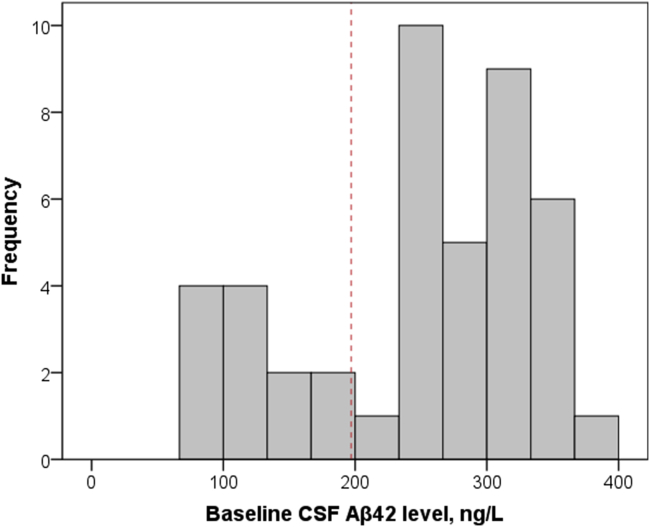


To estimate predictive ability, sensitivity, specificity, positive predictive value, and negative predictive value were calculated. Because of the bimodal distribution of Aβ_42_, this measure was dichotomized using 192 ng/L as a cutoff (Supplementary Fig. 1), which is the same as suggested by Shaw et al. [29]. In addition, Kaplan-Meier survival curves are used for temporal visualization of conversion to dementia diagnosis. Significance level is set to *P* < .05.

## 3 Results

### 3.1 Participants

Forty-four participants (n = 44) were included in the main analyses of the present study, as described in Fig. 1. Demographics, cognitive performance, and CSF biomarker levels are presented in Table 1. During the clinical follow-up period in total, 12 individuals (27%) developed cognitive impairment (Table 1).

### 3.2 Prediction of AD or DLB using baseline CSF biomarkers

**Supplementary Fig. 2:**
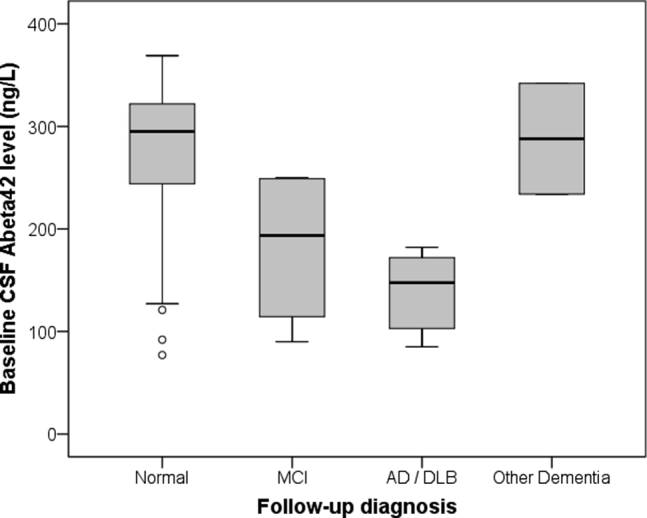


Individuals who developed AD or DLB during follow-up had lower CSF Aβ_42_ levels at baseline compared with the cognitive stable individuals and OD individuals, with MCI at intermediate levels (χ^2^, 12.2; degree of freedom [df], 3; *P* < .01; Supplementary Fig. 2). No differences in T-tau or P-tau levels were seen.

Twelve cognitively healthy individuals (27%) had CSF Aβ_42_ levels at baseline below the cutoff of <192 ng/L [29] and were, hence, interpreted as having pathologic levels. Six individuals in this group (50%) had developed AD (n = 5) or DLB (n = 1) after 9 years of follow-up. In contrast, no one with normal baseline CSF Aβ_42_ levels had developed AD or DLB. Thus, CSF Aβ_42_ predicted development of AD or DLB within 9 years with a sensitivity of 100%, specificity of 84%, positive predictive value of 50%, and negative predictive value of 100%.

**Fig. 2:**
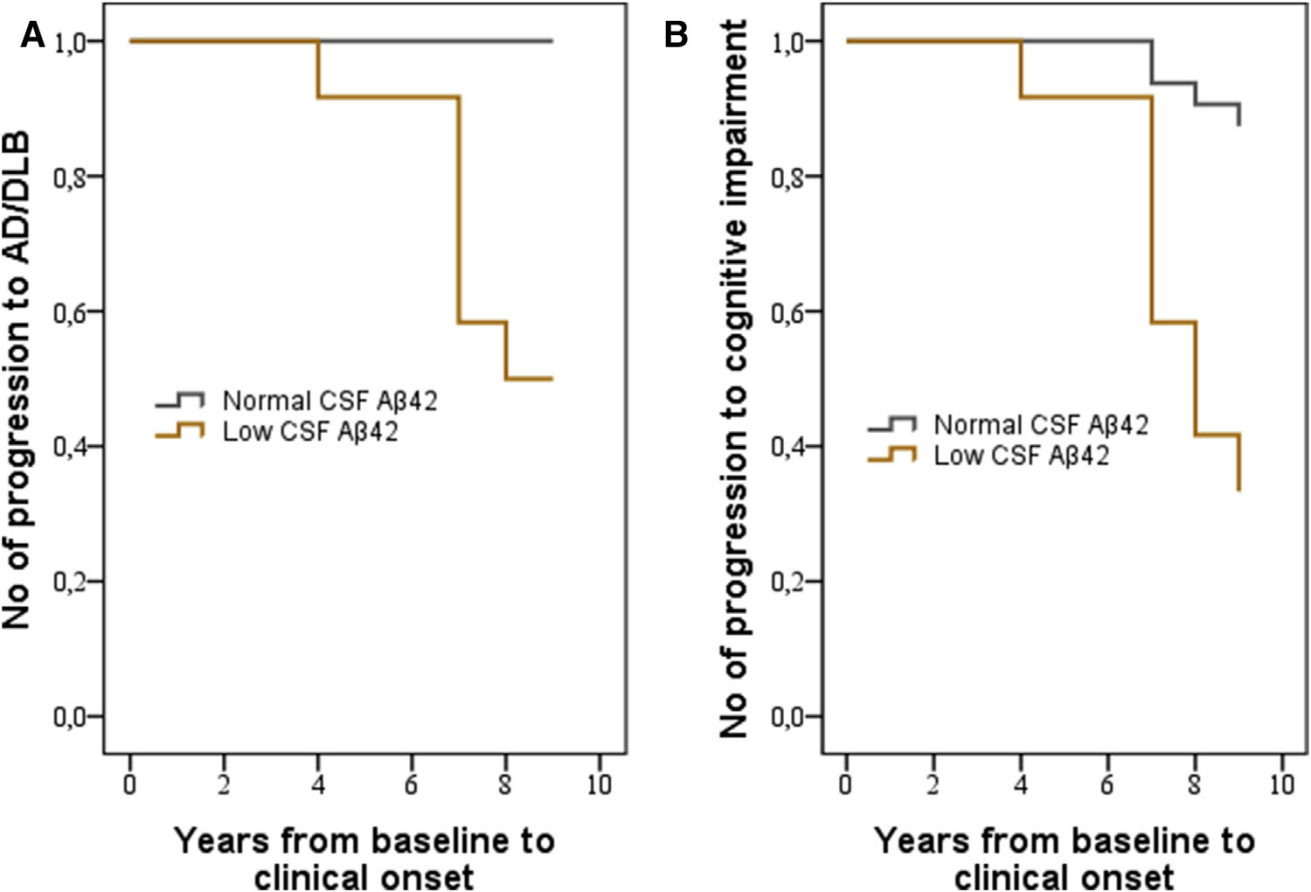
Kaplan-Meier curves for development of cognitive diagnosis depending on CSF Aβ_42_ status (A) Development of Alzheimer's dementia and dementia with Lewy bodies during follow-up in the group with low baseline CSF Aβ_42_ levels compared with the group with normal levels. (B) Development of cognitive impairment (AD, DLB, VaD, or MCI) during follow-up in the group with low baseline CSF Aβ_42_ levels compared with the group with normal levels. n = 44. Abbreviations: CSF, cerebrospinal fluid; Aβ_42_, β-amyloid 42; AD, Alzheimer's disease; DLB, dementia with Lewy bodies; MCI, mild cognitive impairment.

Fig. 2A shows Kaplan-Meier survival curves of low baseline CSF Aβ_42_ levels compared with normal levels for prediction of subsequent development of AD or DLB. Cox regression model revealed that cognitively healthy individuals with low baseline Aβ_42_ levels had an increased risk of subsequent AD or DLB with an HR of 11.9 (95% CI, 1.5–94.2; *P* < .05) for each SD decrease of CSF Aβ_42_ levels when adjusted for age, gender, presence of *APOE* ε4 allele, and baseline delayed word recall. In contrast, CSF tau and P-tau did not predict development of AD/DLB over 9 years and the ratios of CSF Aβ_42_/tau or Aβ_42_/P-tau did not improve the predictive ability compared with CSF Aβ_42_ alone.

### 3.3 Prediction of MCI or dementia using baseline CSF biomarkers

Twelve individuals (27%) developed some form of cognitive impairment during the 9-year follow-up (Table 1). Of these, eight had low CSF Aβ_42_ at baseline (two MCI, five AD, and one DLB) and four had normal baseline levels (two MCI, one vascular dementia, and one other type of dementia). Thus, CSF Aβ_42_ predicted cognitive impairment in general (MCI or dementia) with a sensitivity of 67%, specificity of 88%, positive predictive value of 67%, and negative predictive value of 88%. Cox regression model revealed that cognitively healthy individuals with low baseline Aβ_42_ levels had an increased risk of subsequent cognitive impairment with an adjusted HR of 3.9 (95% CI, 1.8–8.4; *P* < .05) for each SD decrease of CSF Aβ_42_ (Fig. 2B).

### 3.4 Prediction of AD or DLB using baseline cognitive tests

No difference in baseline cognitive test results was seen between the individuals that developed AD/DLB at follow-up compared with those who remained cognitively stable (*P* > .05). However, the group of individuals that developed AD/DLB had significantly lower delayed memory scores already at the 5-year follow-up compared with those in the other groups (χ^2^, 8.46; df, 3; *P* < .05). When added to the Cox regression model, baseline delayed word recall score (ADAS-cog item 3) contributed to predict development of AD and DLB with HR of 2.8 (95% CI, 1.1–6.7; *P* < .05) for each not correctly given word. However, this predictive ability disappeared if CSF Aβ_42_ levels were removed from the model. This is in contrast to CSF Aβ_42_ that remained an independent predictive factor irrespective of delayed word recall. Hence, CSF Aβ_42_ levels is the driving predictive factor 9 years before diagnosis in this cohort, and episodic memory only contributes if amyloid status is taken into account.

### 3.5 Low baseline CSF Aβ_42_ levels and cognitive stability over 9 years

Individual demographics and cognitive follow-up data, for each of the participants with low baseline CSF Aβ_42_ level, are specified in Table 2. Note that 4 of 12 individuals (33%) exhibited no cognitive symptoms and performed well on cognitive testing even after 9 years of follow-up. On the other hand, these four individuals had higher CSF P-tau levels at both baseline (*U* = 96.0; *P* < .001) and at follow-up after 9 years (*U* = 41.5; *P* < .05) compared with the cognitively normal individuals with normal baseline CSF Aβ_42_ levels.

### 3.6 Longitudinal CSF biomarker levels over 9 years

**Fig. 3:**
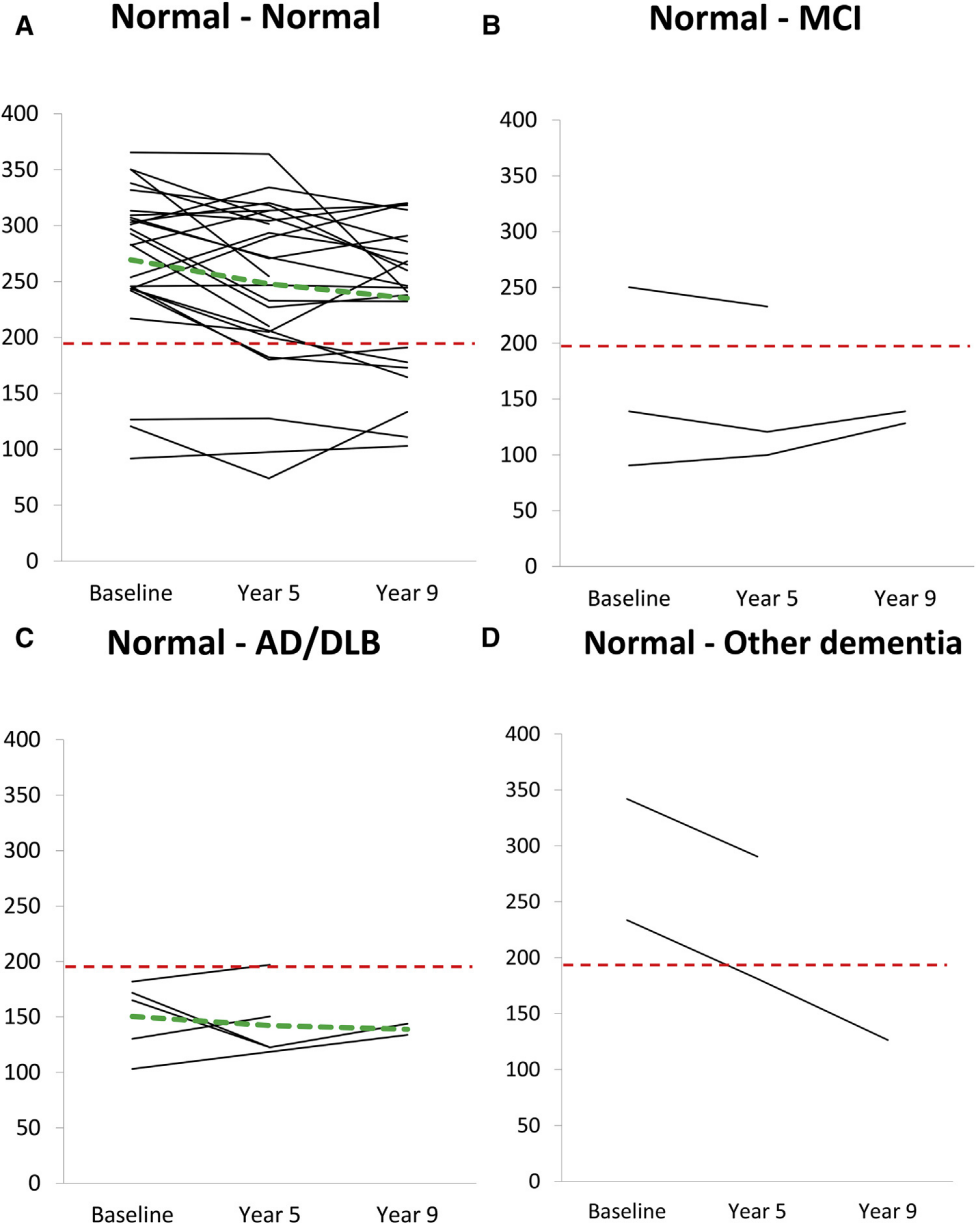
Temporal development of CSF Aβ_42_ levels for each individual divided according to follow-up cognitive diagnoses n = 36 individuals. CSF: all occasions = 23 individuals, baseline + year 5 = 9 individuals, baseline + year 9 = 4 individuals. Cognitive groups: (A) Normal-Normal n = 26 individuals, (B) Normal-MCI n = 3 individuals, (C) Normal-AD/DLB n = 5 individuals, and (D) Normal-other dementia n = 2 individuals. Green dotted line represents change of mean value for each group with more than four participants. Red dotted line indicates cutoff 192 ng/L, suggested by Shaw et al [29]. Abbreviations: CSF, cerebrospinal fluid; Aβ_42_, β-amyloid 42; MCI, mild cognitive impairment; AD, Alzheimer's disease; DLB, dementia with Lewy bodies.

Thirty-six of the individuals with CSF measurements at baseline had at least one repeated CSF acquisition during follow-up period (year 5 or year 9), of which 23 individuals had from all three occasions (Fig. 3 and Supplementary Figs. 3 and 4). On group level, there was a decrease in CSF Aβ_42_ levels (*T* = 85.0; *P* < .05) and an increase in CSF T-tau levels (*T* = 329.5; *P* < .01) between baseline and follow-up after 9 years, whereas CSF P-tau remained stable. Baseline CSF Aβ_42_ levels correlated negatively with baseline CSF P-tau levels (*r*_*s*_ = −0.48, *P* < .001). Baseline CSF P-tau levels also correlated negatively with CSF Aβ_42_ levels at follow-up (*r*_*s*_ = −0.61, *P* < .001). Moreover, baseline CSF P-tau levels were significantly higher in the individuals with low baseline CSF Aβ_42_ when compared with those with normal baseline CSF Aβ_42_ (*U* = 310.0; *P* < .01), but not at follow-up 9 years later.

Low baseline CSF Aβ_42_ levels were observed in 10 of these individuals and no further decrease over time occurred (Fig. 3). Instead, the CSF Aβ_42_ levels were stably decreased in these 10 individuals during follow-up, of which 7 individuals had developed AD/DLB or MCI at the 9-year follow-up visit. In contrast, 5 of the remaining 26 individuals (19%) converted from normal CSF Aβ_42_ levels at baseline to pathologic low levels during follow-up. Compared with those with normal CSF Aβ_42_ levels also at follow-up, these individuals had higher CSF P-tau levels at baseline (*U* = 95.0, *P* < .05) as well as at follow-up after 9 years (*U* = 67.0, *P* < .01), whereas follow-up cognitive test results, age, sex, *APOE* genotype, and education did not differ.

Hence, significantly higher baseline and follow-up CSF P-tau levels were observed in all cognitively stable participants with low CSF Aβ_42_ levels (at baseline or converters during follow-up) compared with those cognitively stable with consistent normal CSF Aβ_42_ levels. Finally, we observe that all individuals with MCI and AD/DLB diagnoses tend to increase over time in CSF T-tau levels even though this was not statistically significant (Supplementary Figs. 3 and 4).

## 4 Discussion

To our knowledge, no previous study has measured CSF biomarkers repeatedly over an extended period during the preclinical phases of sporadic AD, which is needed to be able to determine the trajectories of biomarker changes during the preclinical phase of sporadic AD. In the present study, the data suggest that CSF Aβ_42_ is decreased up to 9 years before dementia onset and does not decrease further during this preclinical phase, i.e. CSF Aβ_42_ has already plateaued down to fully decreased level 9 years before AD dementia onset. Indeed, the current results do actually imply that the decrease in CSF Aβ_42_ occurs more than a decade before onset of sporadic AD/DLB because several cases with low CSF Aβ_42_ at baseline did not develop MCI or dementia during the 9-year follow-up and none of the individuals that converted from normal to low Aβ_42_ levels during follow-up did develop MCI or AD. These data are in agreement with data obtained from studies evaluating CSF Aβ_42_ levels in asymptomatic cases with autosomal dominant AD [1], [5]. The present data extend knowledge obtained from studies showing that CSF Aβ_42_ levels are quite stable during the dementia and MCI phases of AD [30], [31]. The annual incidence rate of CSF Aβ_42_ conversion was 2% in this study, which supports that CSF Aβ_42_ becomes reduced during adulthood rather than childhood/adolescence.

The changes in CSF tau and P-tau might be more subtle than the change in CSF Aβ_42_ during the preclinical stages of AD, and consequently more difficult to reliably detect in CSF [3], [32]. In the present study, development of AD/DLB is not associated with baseline CSF tau or P-tau levels. However, the individuals that developed AD/DLB had a quite slow disease progression rate with relatively low tau levels even at dementia onset. Instead, lower CSF Aβ_42_ levels are associated with higher CSF P-tau levels throughout follow-up. Together, these data imply that CSF P-tau might change as early as Aβ_42_ in preclinical AD, even though the changes are less pronounced compared with those of CSF Aβ_42_. Similar finding has recently been observed in a study examining cases with autosomal dominant AD [5].

Diagnostic methods are needed to accurately detect preclinical AD to be able to recruit individuals with yet only limited neurodegeneration for clinical trials. The increased risk of future development of AD or DLB in cognitively healthy elderly individuals with low CSF Aβ_42_ levels is in agreement with previous studies [9], [10], [11], [12], [14]. However, despite a high negative predictive value (100%), several individuals with low baseline CSF Aβ_42_ levels did not develop AD or DLB during a follow-up period of nearly a decade. In fact, a remarkable one-third (n = 4) of the individuals with low baseline CSF Aβ_42_ levels remained completely cognitive intact (Table 2). We later confirmed pathologic amyloid load in neocortex in all these four individuals at follow-up using [^18^F]flutemetamol PET (Supplementary Material). The study, therefore, highlights the possibility for elderly individuals to harbor amyloid pathology for a long period without development of cognitive dysfunction. Our findings, hence, demonstrate the difficulties of using amyloid markers, such as CSF Aβ_42_ or amyloid PET, alone in the diagnostic workup of preclinical AD. The combination with one or preferably several markers that reflects brain dysfunction or neurodegeneration has been suggested [33], [34], such as with regional brain atrophy [35], [36], regional cerebral hypoperfusion [37], [38], decreased regional glucose metabolism [36], [39], or altered cortical connectivity (through resting state functional magnetic resonance imaging) [40]. To optimize this diagnostic workup will in the future be very important when recruiting individuals with signs of brain amyloid accumulation, but no cognitive symptoms or impairment, for clinical trials evaluating new disease-modifying therapies that might cause significant side-effects. The tolerance for side-effects in such trials should be low if inclusion criteria are used that results in recruitment of population where up to 50% in the placebo group will be free of AD after more than a decade. Therefore, there is an urgent need for multimodal studies in cognitively healthy elderly individuals evaluating different diagnostic algorithms including amyloid biomarkers in combination with different biomarkers that reflect disease stage.

There are limitations to this study. First, the number of participants is too low for extended subgroup analyses. Second, cognitive impairment occurs late in the follow-up. This could indicate that the group at baseline did not represent the entire spectrum of noncognitively impaired individuals in the community. Third, only one participant developed DLB, which prevents statistical analysis of this disease group separately. However, DLB often presents amyloid pathology and presumably lies in the disease spectrum between Parkinson's disease (synucleinopathy) and AD (amyloidopathy) [28]. We, therefore, grouped AD and DLB, but the findings of this study remain also if the DLB participant is removed from this group. Fourth, CT was used for neuroimaging throughout the study, mainly owing to magnetic resonance imaging not being as accessible at the start of the study in 2002. The study was also planned primarily as a CSF biomarker study, in which CT is sufficient to exclude contraindications for lumbar puncture and to evaluate occurrence of clinically relevant cerebral lesions. Nevertheless, the study has invaluable strengths. We describe within-person changes in CSF biomarkers over a decade in healthy elderly individuals. Because a subgroup developed AD, we could also study these within-person changes during the preclinical (asymptomatic) stages of AD. All conversions to dementia occurred between follow-up year 5 and year 9 and would not have been identified with shorter follow-up. Hence, secondary end points are replaced by primary end points (i.e. dementia diagnosis). Finally, 44 of 54 individuals (81%) had clinical follow-up evaluation, which is very high in the light of 9 years of follow-up in an older population. With the medical record examination of the deceased, only two individuals (4%) of the included subjects lack 9-year follow-up. Hence, the results reflect the actual state of this entire study population.

## 5 Conclusions

Low CSF Aβ_42_ levels predict development of AD at least 9 years before dementia onset with a very high negative predictive value. Data obtained from the repeated CSF measurements imply that CSF Aβ_42_ levels have decreased and reached a plateau already a decade before clinical onset of dementia in sporadic AD. Surprisingly, many individuals can harbor brain amyloid accumulation over a decade without any signs of cognitive deterioration, which will clearly implicate how CSF biomarkers are used to identify preclinical AD in future interventional therapeutic trials.

**Research in context**

1. Systematic review: Traditional sources (such as PubMed) were reviewed with focus on predicting sporadic dementia in cognitively healthy individuals. A handful publications have investigated these elongated preclinical stages, although very few with follow-up exceeding 5 years. Instead, current evidence is mainly derived from evaluating autosomal dominant Alzheimer's disease (AD).
2. Interpretation: Our findings improve the understanding of how cerebrospinal fluid AD-biomarkers can be interpreted in absence of cognitive symptoms. They align with current hypothesis that β-amyloid is an early marker of AD but also confirm difficulties of interpreting their predictive relevance on individual level. This could implicate their use for identifying preclinical AD in future therapeutic trials.
3. Future directions: The article elucidates the need for long follow-up in preclinical AD studies. This to clarify (1) how long individuals can harbor brain amyloid accumulation without cognitive impact and (2) which biomarker combination that correctly identifies preclinical AD with impending risk of dementia conversion.

## Figures and Tables

**Table 1:** Group characteristics at baseline and at follow-up year 5 and year 9, including follow-up diagnoses

Characteristics	Baseline	Follow-up year 5	Follow-up year 9
Baseline demographics			
Number (n)	44		
Sex (F/M)	29/15		
Age (y)	72 (65–78)		
Education (y)	11 (9–15)		
*APOE* ε4 (n) (homo-/heterozygous)	1/14		
Follow-up time (mo)	111 (110–112)		
Cognitive tests			
MMSE (points)	30.0 (29.0–30.0)	29.0 (27.0–29.0)	28.0 (26.0–29.0)
AQT (s)∗	62.0 (52.5–69.0)†	61.0 (53.5–74.5)	67.0 (56.5–84.0)
Delayed memory (ADAS), correct words of 10	8.0 (7.0–9.0)	8.0 (7.0–9.0)	8.0 (6.0–9.0)
CSF biomarkers	n = 44	n = 32	n = 27‡
Aβ_42_	252.0 (174.5–312.0)	230.0 (180.5–292.5)	238.0 (139.0–275.0)
T-tau	67.5 (55.5–92.0)	74.0 (58.0–99.0)	79.0 (58.0–111.0)
P-tau	27.0 (21.5–43.5)	35.0 (21.0–50.0)	29.0 (22.0–41.0)
Follow-up diagnosis, n			n = 44 (53)§
Normal			32 (39)
Mild cognitive impairment			4 (5)
Alzheimer's dementia			5 (5)
Lewy body dementia			1 (1)
Vascular dementia			1 (1)
Other dementia			1 (1)

Abbreviations: *APOE*, apolipoprotein E; MMSE, mini-mental state examination; AQT, a quick test; ADAS, Alzheimer's disease assessment scale; CSF, cerebrospinal fluid; Aβ_42_, β-amyloid 42; T-tau, total tau; P-tau, phosphorylated tau.

NOTE. Median values with 25th–75th interquartile range within brackets.

∗ Color-form version of AQT is stated (reference value <70 s).

† AQT was not performed at baseline and, therefore, values from follow-up year 3 are given.

‡ Not all have CSF measurement from year 5, which results in 36 participants with two CSF assessments and 23 participants with all three CSF assessments. CSF levels are given in ng/L.

§ Numbers within brackets include premortem diagnosis for participants who deceased during follow-up and that were included in the Cox regression model.

**Table 2:** Individual follow-up cognitive diagnoses, performances, and baseline demographics for the subgroup of participants with low baseline CSF Aβ_42_ levels

Participant	Follow-up year 9							Demography			Baseline				
Diagnosis	Year of diagnosis	Age	MMSE (points)	Delayed word recall (correct of 10)	AQT (s)∗	Clock test (correct)	Sex	Education (y)	*APOE* genotype	Aβ_42_ (ng/L)	T-tau (ng/L)	P-tau (ng/L)	MMSE (points)	Delayed word recall (correct of 10)
A	AD	2012	84	19	3	91	Yes	Female	7.0	3/4	130	97	49	30	7
B	AD	2012	93	21	2	83	No	Female	7.0	3/4	103	102	61	29	9
C	AD	2007†	80	21	0	150	No	Male	9.0	3/4	172	45	26	29	8
D	AD	2010	92	21	0	176	No	Female	13.0	3/3	182	33	11	30	5
E	AD	2007†	83	12	0	184	No	Female	9.0	3/4	85	157	55	30	6
F	DLB	2010†	82	30	7	80	No	Male	18.0	3/3	165	94	45	30	8
G	MCI	2012	83	25	6	121	No	Female	8.0	3/3	90	62	25	30	10
H	MCI	2012	91	28	7	146	Yes	Male	12.5	3/4	139	82	34	28	8
I	Normal	NA	88	29	7	68	Yes	Male	16.5	3/3	77	70	62	30	8
J	Normal	NA	81	28	6	50	Yes	Female	15.0	3/3	127	145	74	28	7
K	Normal	NA	81	30	9	71	Yes	Female	7.0	3/4	121	139	69	30	10
L	Normal	NA	72	29	10	40	Yes	Male	10.0	3/3	92	63	45	27	9

Abbreviations: CSF, cerebrospinal fluid; Aβ_42_, β-amyloid 42; MMSE, mini-mental state examination; AQT, a quick test; APOE, apolipoprotein E; T-tau, total tau; P-tau, phosphorylated tau; AD, Alzheimer's disease; DLB, dementia with Lewy bodies; MCI, mild cognitive impairment; NA, not applicable.

NOTE. Year of diagnosis state when the participant was given its first cognitive diagnosis.

∗ Color-form version of AQT is stated (reference value <70 s).

† Year of preceding MCI diagnosis.
