# Supplementary material for: Longitudinal cerebrospinal fluid biomarker measurements in preclinical sporadic Alzheimer's disease: A prospective 9-year study
Source: Alzheimers Dement (Amst). 2015 Oct 9;1(4):403–11. doi: 10.1016/j.dadm.2015.09.002 (PMC4879483; doi:10.1016/j.dadm.2015.09.002)
Supplement: Supplementary Material [file mmc1.docx]

**Supplementary Materials**

**Methods**

*Amyloid PET scanning and analysis*

[^18^F]Flutemetamol PET was used, as previously described [1], in those cases with low CSF ab42 levels at baseline and no cognitive symptoms or deficits after 9-10 years of follow-up. Briefly, [^18^F]flutemetamol was manufactured using a TracerLab-FXF-N chemistry platform (GE Healthcare) [2]. PET/CT scanning of the whole brain was conducted using a Philips Gemini TF 16 scanner. PET sum images from approximately 90-120 min post injection were generated describing the average uptake of [^18^F]flutemetamol over this time span. MRI data were not involved since they do not improve the quantification of [^18^F]flutemetamol data [3]. The images were analyzed using the software NeuroMarQ provided by GE Healthcare. A volume of interest (VOI) template for different cortical and subcortical regions was applied. A global neocortical composite region was used [3]. The standardized uptake value ratio (SUVR) was defined as the regional tracer uptake in a VOI, normalized for the mean uptake in the cerebellar cortex. In a previous study we have found that a cut off the composite SUVR of [^18^F]flutemetamol of 1.42 separates those with normal scans to those with abnormal scans [1].

**References**

[1] Palmqvist S, Zetterberg H, Blennow K, Vestberg S, Andreasson U, Brooks DJ, et al. Accuracy of brain amyloid detection in clinical practice using cerebrospinal fluid beta-amyloid 42: a cross-validation study against amyloid positron emission tomography. JAMA neurology. 2014;71:1282-9.

[2] Nelissen N, Van Laere K, Thurfjell L, Owenius R, Vandenbulcke M, Koole M, et al. Phase 1 study of the Pittsburgh compound B derivative 18F-flutemetamol in healthy volunteers and patients with probable Alzheimer disease. Journal of nuclear medicine : official publication, Society of Nuclear Medicine. 2009;50:1251-9.

[3] Lundqvist R, Lilja J, Thomas BA, Lotjonen J, Villemagne VL, Rowe CC, et al. Implementation and validation of an adaptive template registration method for 18F-flutemetamol imaging data. Journal of nuclear medicine : official publication, Society of Nuclear Medicine. 2013;54:1472-8.

**Figure legends**

**Suppl fig 1** Distribution of baseline CSF Aβ_42_ levels. Red dotted line indicates the cut-off <192 ng/L.

**Suppl fig 2** Box-plot of baseline CSF Aβ_42_ levels in relation to follow-up diagnosis after nine years. Median values and 25^th^-75^th^ interquartile ranges are shown. Circles define outliers between 1.5 and 3 times the interquartile range. N = 44.

**Suppl fig 3** Temporal development of CSF t-tau levels for each individual divided according to follow-up cognitive diagnoses.

N = 36 individuals. CSF: all occasions = 23 individuals, baseline + year 5 = 9 individuals, baseline + year 9 = 4 individuals. Cognitive groups: Normal-Normal n = 26 individuals, Normal-MCI n = 3 individuals, Normal-AD/DLB n = 5 individuals, Normal-other dementia n = 2 individuals. Green dotted line represents change of mean value for each group with more than 4 participants.

**Suppl fig 4** Temporal development of CSF p-tau levels for each individual divided according to follow-up cognitive diagnoses.

N = 36 individuals. CSF: all occasions = 23 individuals, baseline + year 5 = 9 individuals, baseline + year 9 = 4 individuals. Cognitive groups: Normal-Normal n = 26 individuals, Normal-MCI n = 3 individuals, Normal-AD/DLB n = 5 individuals, Normal-other dementia n = 2 individuals. Green dotted line represents change of mean value for each group with more than 4 participants.
