# Supplementary figures and images for: Longitudinal cerebrospinal fluid biomarker measurements in preclinical sporadic Alzheimer's disease: A prospective 9-year study
Source: Alzheimers Dement (Amst). 2015 Oct 9;1(4):403–11. doi: 10.1016/j.dadm.2015.09.002 (PMC4879483; doi:10.1016/j.dadm.2015.09.002)

# Normal - Normal

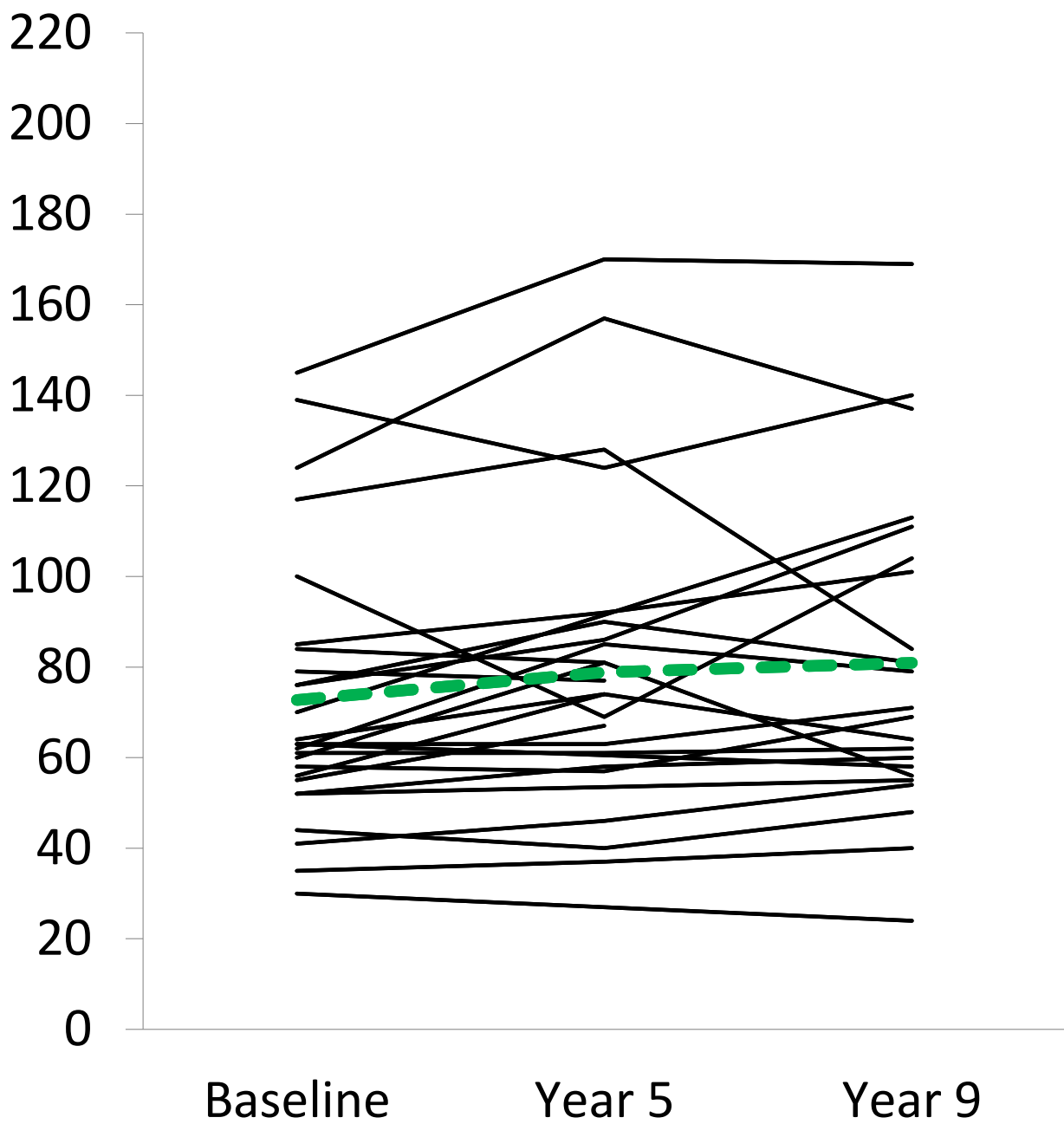

Supplement: Supplementary Fig. 3a [file mmc2.pdf]

# Normal - MCI

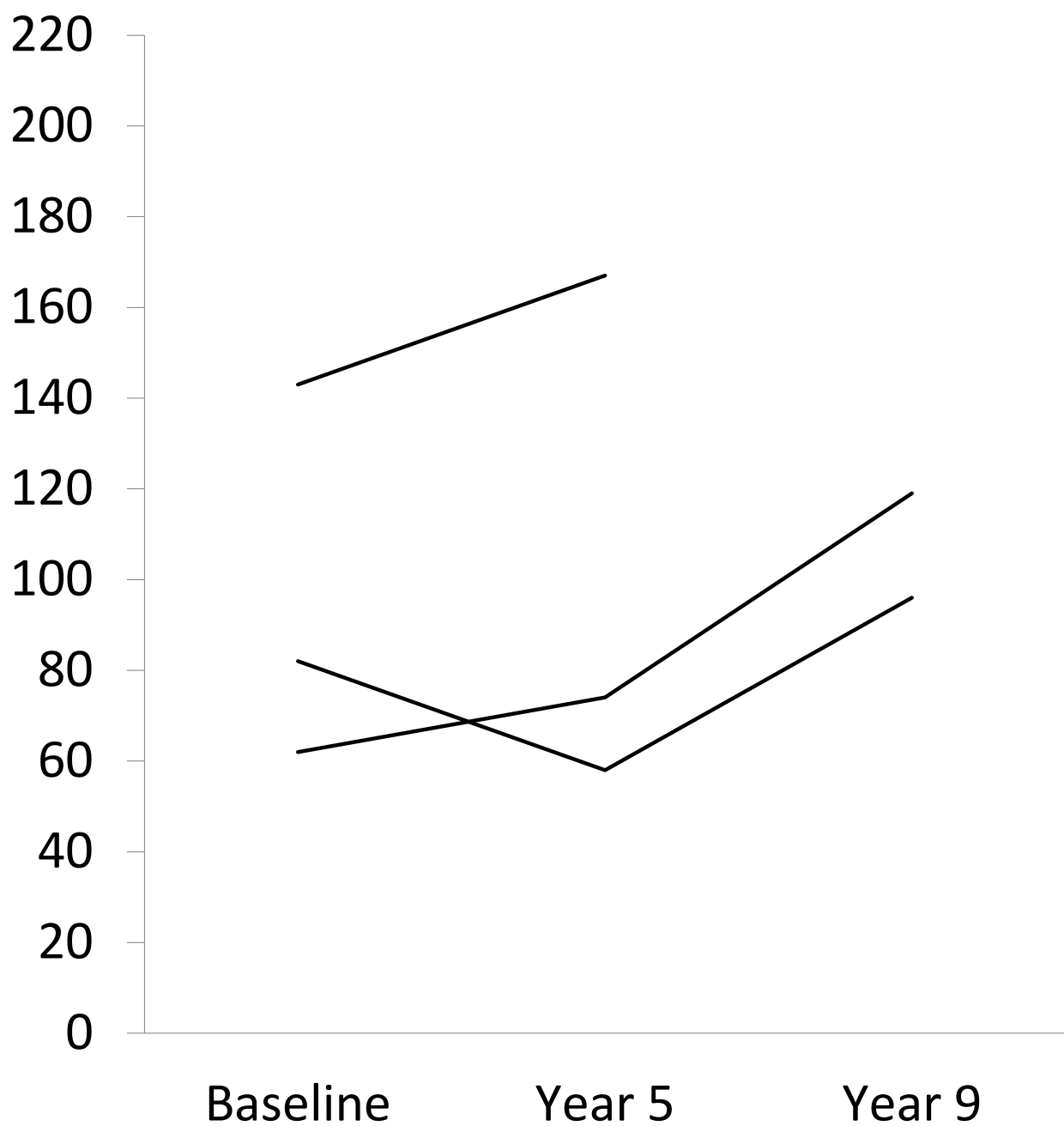

Supplement: Supplementary Fig. 3b [file mmc3.pdf]

# Normal - AD/DLB

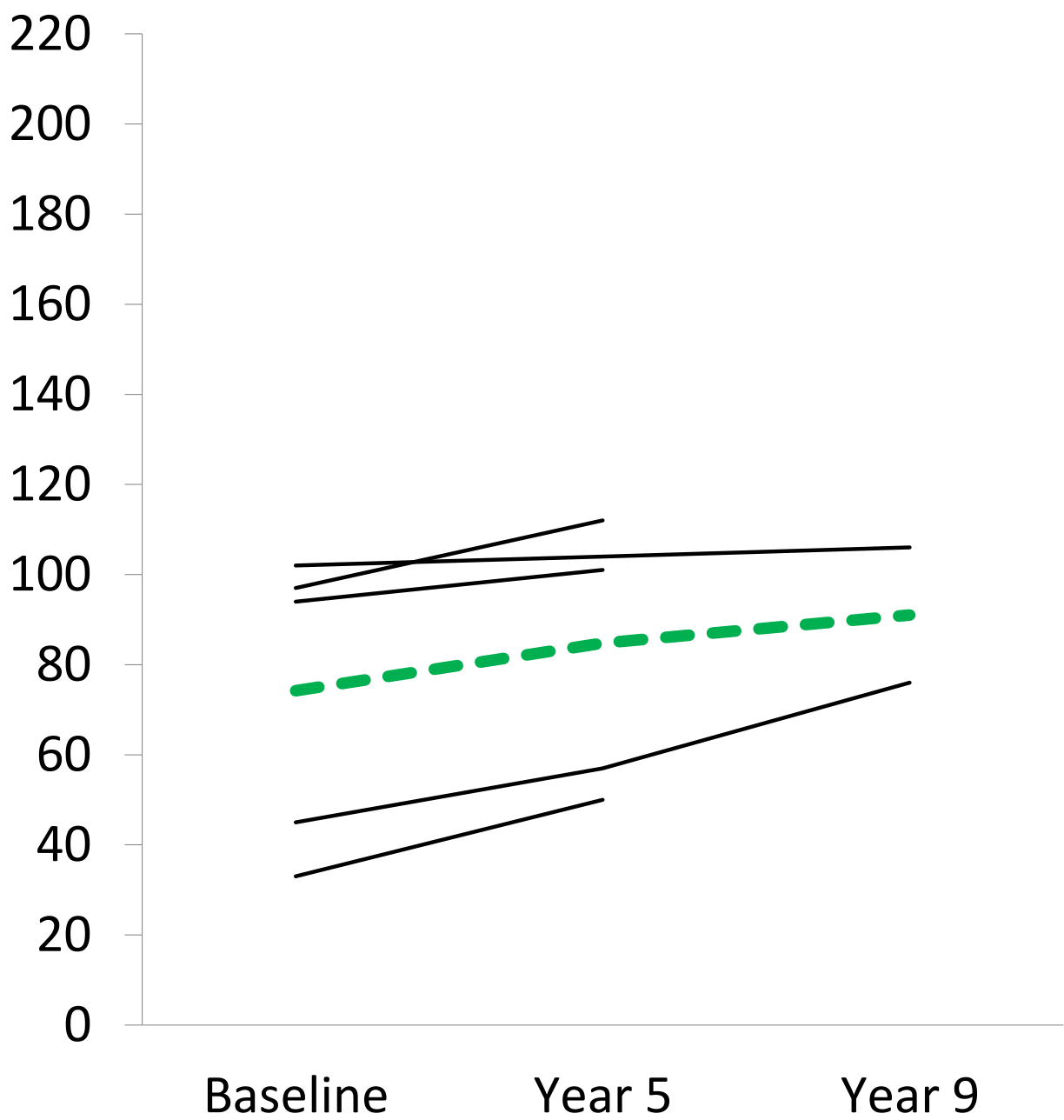

Supplement: Supplementary Fig. 3c [file mmc4.pdf]

# Normal - Other dementia

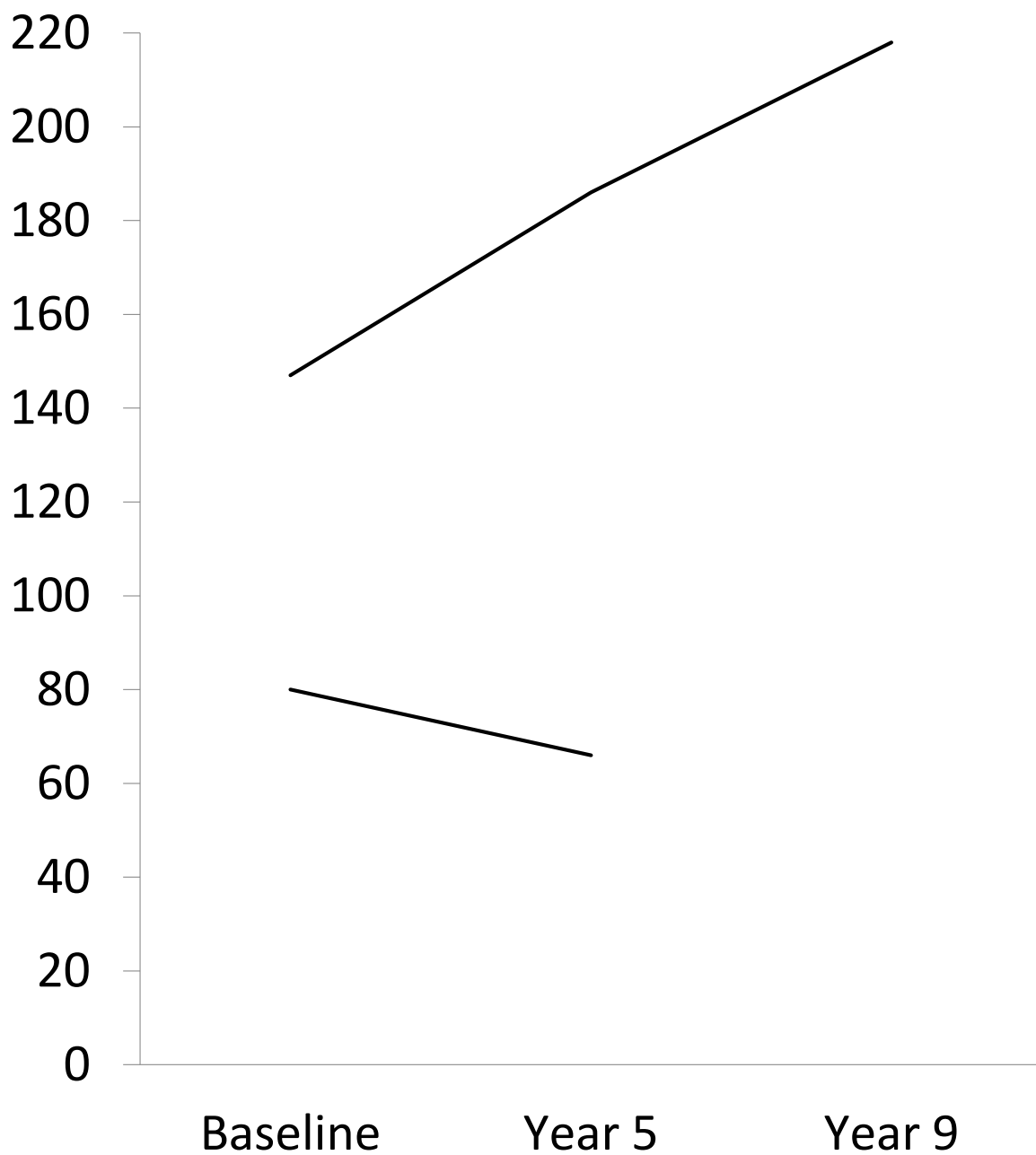

Supplement: Supplementary Fig. 3d [file mmc5.pdf]

# Normal - Normal

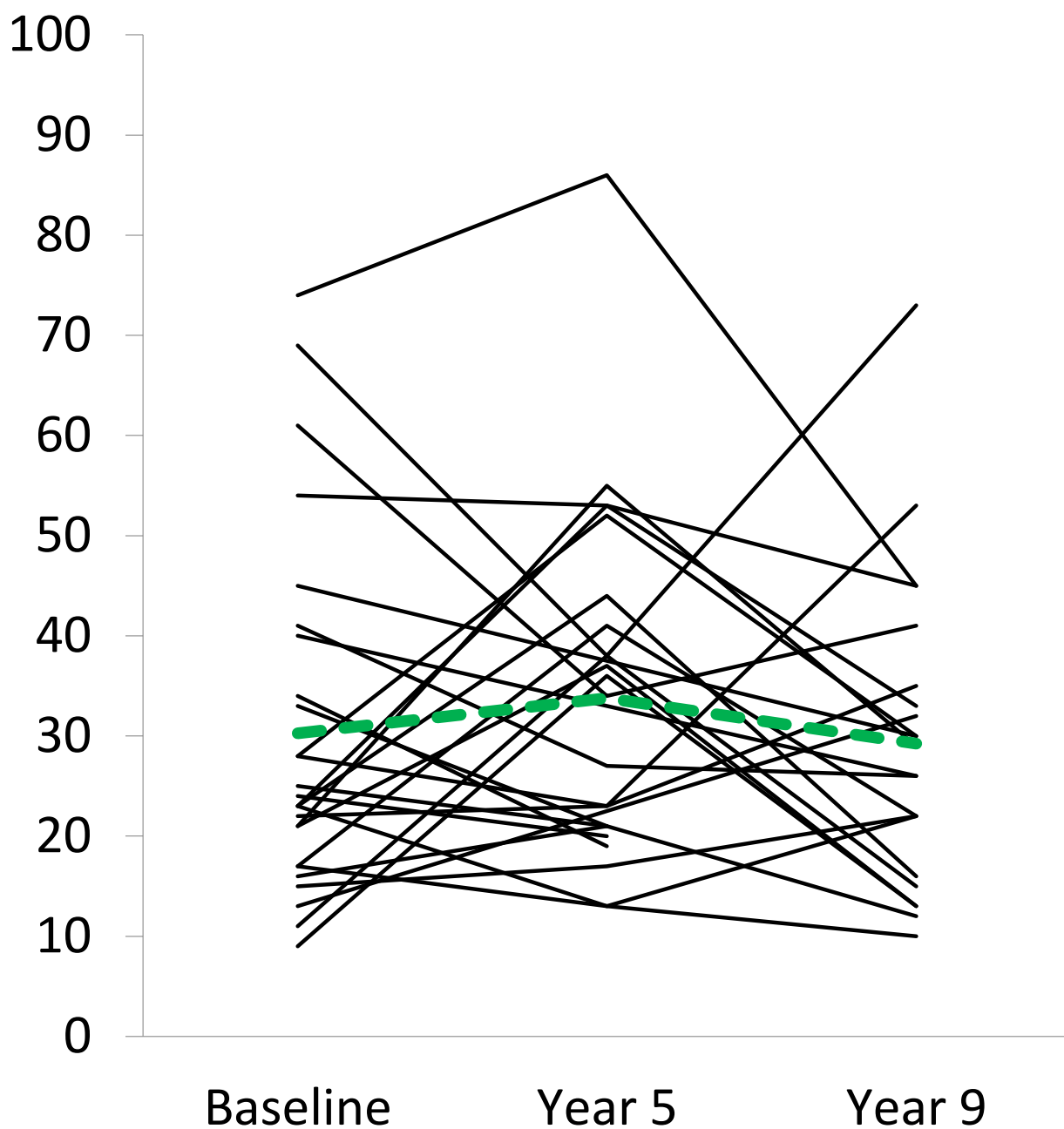

Supplement: Supplementary Fig. 4a [file mmc6.pdf]

# Normal - MCI

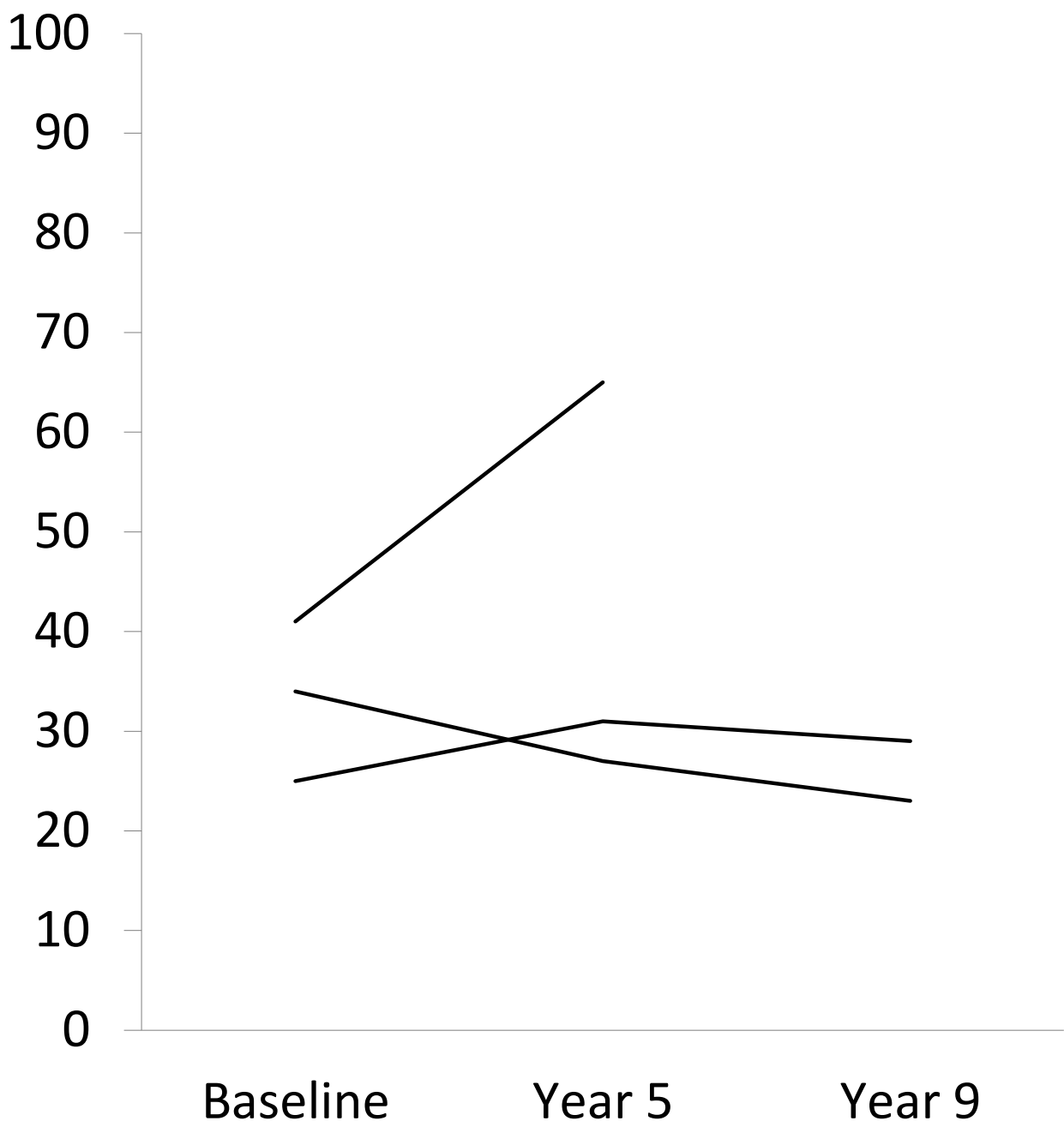

Supplement: Supplementary Fig. 4b [file mmc7.pdf]

# Normal - AD/DLB

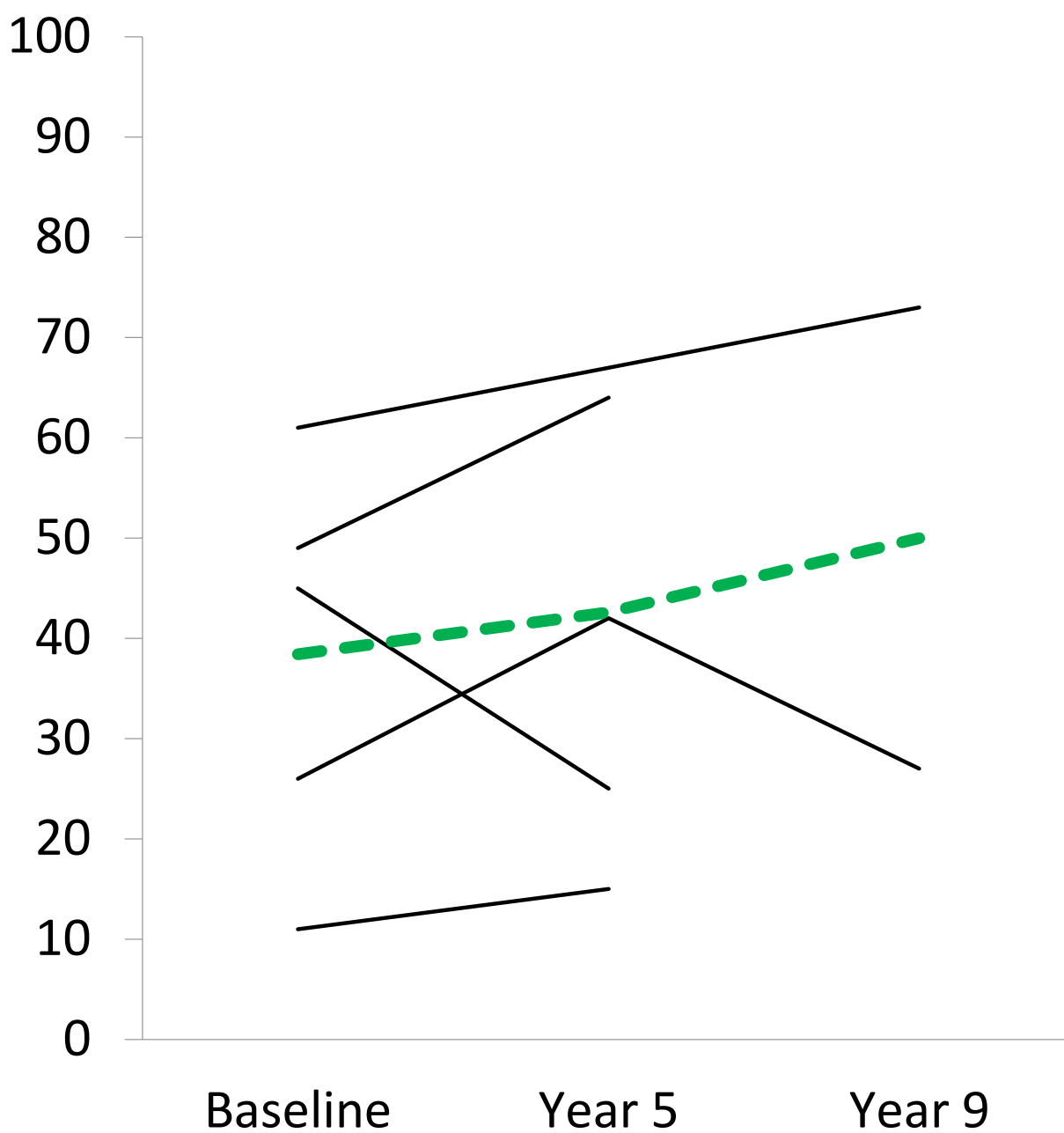

Supplement: Supplementary Fig. 4c [file mmc8.pdf]

## Normal - Other dementia

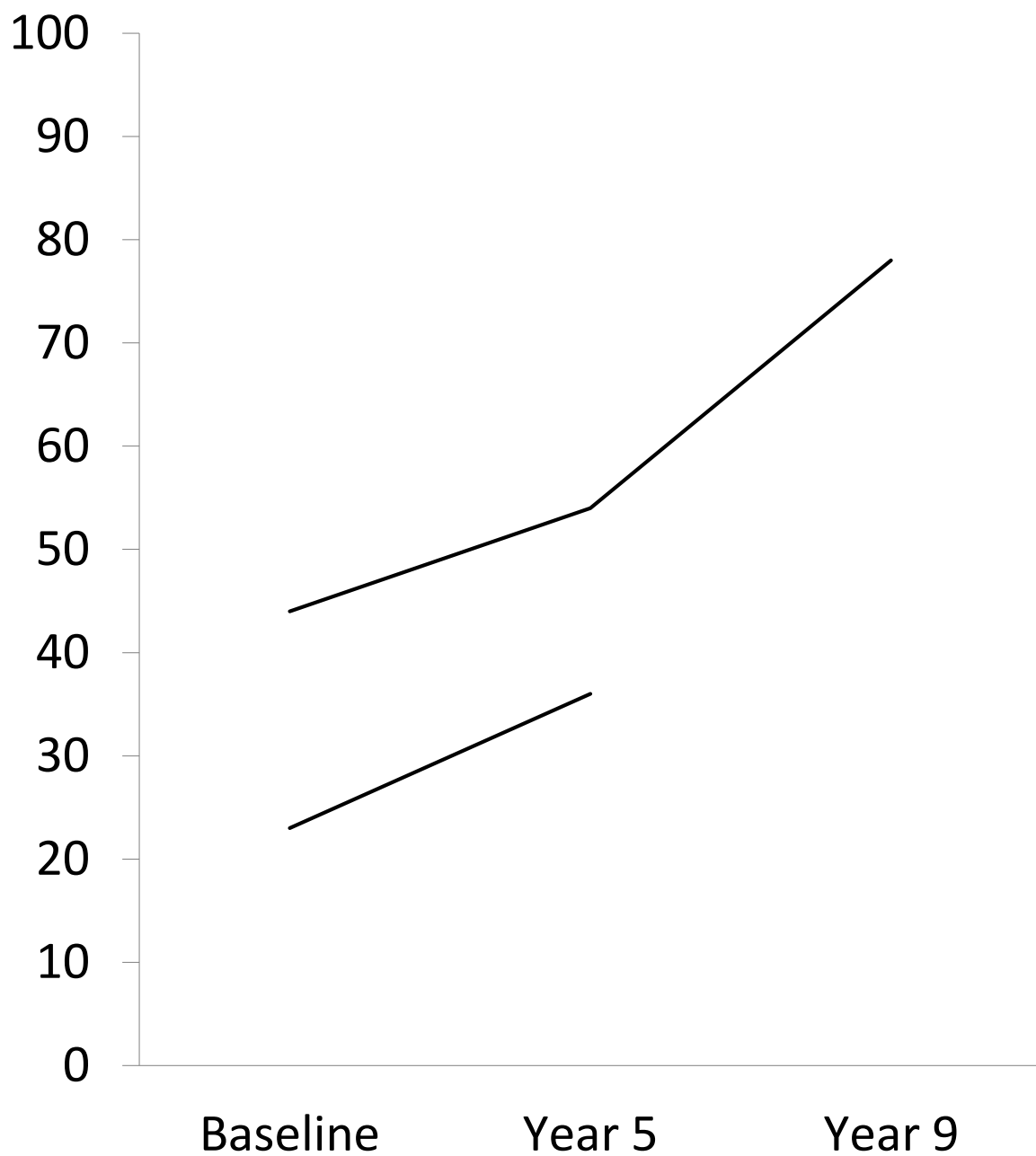

Supplement: Supplementary Fig. 4d [file mmc9.pdf]
